# Genetic variants in the vitamin D pathway genes are predictors of the risk of diabetic kidney disease in Central Asian Kazakhstani cohort with type 2 diabetes

**DOI:** 10.3389/fmed.2025.1630725

**Published:** 2025-07-10

**Authors:** Binura Taurbekova, Abay Tursunov, Aida Kabibulatova, Alimzhan Muxunov, Assel Issabayeva, Kuralay Atageldiyeva, Kamilya Sydykova, Aigul Durmanova, Akbayan Markabayeva, Andrey Starodubov, Kymbat Mukhtarova, Wassim Y. Almawi, Antonio Sarria-Santamera

**Affiliations:** ^1^Department of Biomedical Sciences, School of Medicine, Nazarbayev University, Astana, Kazakhstan; ^2^Department of Medicine, School of Medicine, Nazarbayev University, Astana, Kazakhstan; ^3^Department of Medicine, “University Medical Center” Corporate Fund, Astana, Kazakhstan; ^4^Department of Biology, School of Sciences and Humanities, Nazarbayev University, Astana, Kazakhstan; ^5^Medical Center Hospital of the President’s Affairs Administration of the Republic of Kazakhstan, Astana, Kazakhstan; ^6^“B.B.NURA” Hospitals Group, Astana, Kazakhstan; ^7^Science Faculty of Tunisia, Université de Tunis El Manar, Tunis, Tunisia

**Keywords:** CYP27A1, CYP2R1, GC, diabetic nephropathy, polymorphisms, type 2 diabetes mellitus

## Abstract

**Introduction:**

Diabetic kidney disease (DKD) is a common microvascular complication of type 2 diabetes mellitus (T2DM). Given vitamin D’s roles in glucose metabolism and renal function, this study investigated associations between common variants in vitamin D pathway genes (*CYP27A1*, *CYP2R1*, *GC*) and DKD risk.

**Methods:**

This case–control study included 170 patients with DKD, 157 patients without DKD, and 118 normoglycemic healthy controls. Four single nucleotide polymorphisms (SNPs) in the *CYP27A1* (rs17470271), *CYP2R1* (rs1074165), and *GC* (rs4588 and rs7041) were genotyped using real-time PCR with defined clusters.

**Results:**

The *CYP27A1* rs17470271 T/T genotype was significantly associated with a reduced risk of DKD under the recessive model (AOR = 0.32, 95% CI: 0.11–0.93). A similar protective association for *CYP27A1* rs17470271 T/T genotype was observed under the codominant model (OR = 0.34, 95% CI: 0.12–0.99), although this did not remain statistically significant after adjustment. Likewise, the *GC* rs4588 T/T genotype was strongly associated with a decreased risk of DKD under the recessive (AOR = 0.30, 95% CI: 0.10–0.88) and codominant (AOR = 0.28, 95% CI: 0.09–0.85) models. However, haplotype analysis revealed contrasting findings, with the *GC* haplotype carrying the rs4588 G and rs7041 C alleles being associated with an increased risk of DKD compared with healthy controls.

**Discussion:**

These findings suggest that individual variants in vitamin D pathway genes may serve as potential genetic markers for DKD risk stratification. In addition, haplotype analysis may offer complementary insight into genetic contributions to disease susceptibility.

## 1 Introduction

Diabetic kidney disease (DKD) affects approximately 40% of individuals with diabetes and is a leading cause of end-stage renal disease (ESRD) worldwide (1). The pathogenesis of DKD represents a complex interplay of metabolic dysregulation, chronic inflammation, progressive fibrosis, and hemodynamic alterations, influenced by underlying genetic predisposition and epigenetic modifications (2). Ongoing efforts have been made to identify specific factors and pathways linked to DKD onset and progression. Of these, vitamin D has emerged as a strong candidate, owing to its dual role in regulating glucose homeostasis and renal physiological processes.

Vitamin D was traditionally recognized for its canonical role in calcium-phosphate homeostasis and bone mineralization (3). However, growing evidence confirms broader metabolic effects for vitamin D, especially in glucose metabolism and insulin regulation (4). Vitamin D has been shown to preserve pancreatic *β*-cell function, enhance insulin secretion, (5), and improve peripheral insulin sensitivity (6). It also plays a key role in renal physiology by suppressing the renin-angiotensin system (RAS) (7, 8), maintaining renal cellular integrity (9), modulating immune responses (10), and inhibiting fibrotic processes (11). Given these diverse roles in metabolic and renal processes, genetic variation in vitamin D pathway components may contribute to susceptibility to DKD.

The effects of vitamin D involve a multi-step interaction of several genes, including *CYP27A1*, *CYP2R1*, and *GC*. *CYP27A1* (cytochrome P450 family 27 subfamily A member 1) encodes the mitochondrial cytochrome P450 with a 25-hydroxylase activity that contributes to hepatic vitamin D_3_ metabolism (12) and may also support its 25-hydroxylation in extrahepatic tissues (13). CYP2R1 (cytochrome P450 family 2 subfamily R member 1), considered the principal hepatic 25-hydroxylase, catalyzes the conversion of both vitamin D₂ and D₃ into 25-hydroxyvitamin D [25(OH)D], the main circulating form of vitamin D (14). Genetic variations in *CYP2R1* were shown to alter circulating 25(OH)D levels (14, 15), modify protein function and enzymatic activity (14), and potentially affect the downstream vitamin D signaling pathways. The *GC* (group-specific component) gene encodes vitamin D-binding protein (DBP), a critical transporter of circulating 25(OH)D and the biologically active form, 1,25-dihydroxyvitamin D [1,25(OH)_2_D] (16). Genetic variants in the *GC* gene influence serum concentrations of 25(OH)D (17), the bioavailability of vitamin D (18), and individual responses to vitamin D supplementation (19).

Together, these genetic determinants create distinctive patterns of vitamin D metabolism among individuals, potentially driving susceptibility to multiple complex disorders. Prior studies have established associations between certain vitamin D pathway polymorphisms, particularly in *GC* (e.g., rs4588, rs7041, rs2282679) and *CYP2R1* (e.g., rs10741657, rs12794714, rs1993116, rs10766197), and metabolic syndrome and type 2 diabetes mellitus (T2DM) (20–23). Given that DKD is a major microvascular complication of T2DM (8), genetic variants implicated in broad metabolic disturbances may also contribute to DKD susceptibility. Despite growing evidence linking vitamin D pathway genes with metabolic diseases (20–23), the role of specific variants in *CYP27A1*, *CYP2R1*, and *GC* in the susceptibility and progression of DKD remains largely unexplored, underscoring the need for further investigations.

Given this background, we selected representative SNPs in *CYP27A1*, *CYP2R1*, and *GC* for investigation based on their biological relevance and their location within functionally important or potentially regulatory regions. rs17470271, a variant within *CYP27A1*, is located in an intronic region (24) and may influence gene regulation despite not directly altering the coding sequence, potentially affecting the activity of the mitochondrial cytochrome P450 enzyme it encodes. Given the central role of *CYP2R1* in vitamin D metabolism, rs1074165, although not functionally characterized, was included as an exploratory variant due to its location within this key gene, as unannotated intragenic variants may still have regulatory relevance and warrant investigation. rs4588 and rs7041, two missense variants in exon 11 of the *GC* gene, were included as functionally relevant candidates due to their established role in defining DBP isoforms (*Gc*-1 F, *Gc-*1 S, and *Gc-*2), which differ in their binding affinities for 25-hydroxyvitamin D₃ [25(OH)D₃] (*Gc*-1 F > *Gc*-1 S > *Gc*-2) (25), thereby modulating vitamin D transport and bioavailability. These variants also influence DBP’s structural integrity, interaction potential, and functional stability (25), further supporting their relevance in assessing DKD susceptibility. The selected variants in the *CYP27A1*, *CYP2R1*, and *GC* genes have not been extensively investigated in relation to DKD, particularly in Central Asian populations, whose unique genetic admixture may influence susceptibility patterns.

Kazakhstan is a multi-ethnic country in Central Asia, with a population comprising diverse genetic backgrounds (26). Among these, the ethnic Kazakh group forms the majority and represents a genetically distinct population with an admixture of Asian and European ancestral lineage (27, 28). This genetic diversity may give rise to population-specific factors influencing disease susceptibility, which are not captured in homogeneous cohorts and remain understudied in global research.

This study investigated the association between polymorphisms in *CYP27A1* (rs17470271), *CYP2R1* (rs1074165), and *GC* (rs4588, rs7041) and DKD risk among Kazakhstani individuals with T2DM, highlighting population-specific gene-disease interactions in vitamin D metabolism.

## 2 Materials and methods

### 2.1 Study subjects

This case–control study enrolled 445 participants, comprising 327 individuals with T2DM (T2DM group) and 118 normoglycemic healthy controls. The T2DM group was further stratified into patients with DKD (DKD group; *n* = 170) and 157 T2DM cases without DKD (non-DKD group). Within the DKD group, 64 patients with DKD were not undergoing dialysis, while 106 patients presented with ESRD and were on hemodialysis. Participants were recruited from July 2023 to December 2024 at the University Medical Center (Astana), the Medical Center Hospital of the President’s Affairs Administration (Astana), Olymp Laboratories (Astana), and the «B.B.NURA.» Hospitals Group (multiple sites across Astana, Shymkent, East Kazakhstan, and Pavlodar regions). A convenience sampling strategy was employed, enrolling eligible participants during routine clinical visits at the participating sites.

The diagnosis of T2DM was confirmed as per the American Diabetes Association guidelines, defined by any of the following criteria: fasting plasma glucose (FPG) ≥ 7.0 mmol/L, 2-h plasma glucose during oral glucose tolerance test (OGTT) ≥ 11.1 mmol/L, hemoglobin A1C (HbA1c) ≥ 6.5%, or random plasma glucose ≥ 11.1 mmol/L (29). Albumin-creatinine ratio (ACR) ≥ 30 mg/g and/or an estimated glomerular filtration rate (eGFR) < 60 mL/min/1.73m^2^ defined non-dialysis DKD. The eGFR was calculated using the Chronic Kidney Disease Epidemiology Collaboration (CKD-EPI) creatinine equation (30). In dialysis patients, DKD diagnosis relied on a history of proteinuria and the presence of diabetic retinopathy, with other potential causes of renal damage excluded.

T2DM participants were included if they were between 18 and 75 years of age and had a confirmed diagnosis of T2DM for at least 10 years. Exclusion criteria included gestational and secondary forms of diabetes (steroid-induced, pancreatic) and glomerular/tubulointerstitial kidney diseases. Healthy controls self-reported lack of diabetes and chronic kidney disease (CKD), although no laboratory testing was conducted to confirm glycemic status or kidney function.

Comprehensive demographics and biodata of participants were gathered by trained personnel through structured interviews, following an internally developed protocol to ensure consistency in data collection. Height, weight (for body mass index [BMI] calculation), waist and hip circumference (for waist-to-hip ratio) were taken during the visit. Medical history was self-reported by patients and included arterial hypertension, cardiovascular and cerebrovascular diseases, bronchial asthma, chronic obstructive pulmonary disease (COPD), and long-term medication use. BMI classifications were made using WHO-adapted guidelines for Asian populations: normal weight (18.5–22.9 kg/m^2^), overweight (23.0–27.4 kg/m^2^), and obese (≥27.5 kg/m^2^) (31). Smoking status was aligned with established criteria as current (≥ 100 lifetime cigarettes and currently smoking at the time of interview), former (≥ 100 lifetime cigarettes with smoking cessation), and never smokers (< 100 lifetime cigarettes) (32).

### 2.2 Ethics approval

The study was conducted in accordance with the Declaration of Helsinki II, and was approved by the Nazarbayev University Institutional Research Ethics Committee (Approval No. 650/23112022, dated January 10, 2023). All participants received information on the voluntary and anonymous nature of the study and signed an informed consent form before inclusion.

### 2.3 Single nucleotide polymorphism genotyping procedures

Genomic DNA was extracted from peripheral venous blood using the Wizard Genomic DNA Purification Kit according to the manufacturer’s instructions (Promega, Madison, WI). DNA quantity and purity were assessed using a NanoDrop 2000 spectrophotometer (Thermo Fisher Scientific, Wilmington, DE). Genotyping of the *GC* rs4588 and rs7041, *CYP2R1* rs1074165, and *CYP27A1* rs17470271 single-nucleotide polymorphisms (SNPs) was performed using TaqMan® SNP Genotyping Assays on QuantStudio 6 and 7 real-time polymerase chain reaction (PCR) systems, following the manufacturer’s protocols (Applied Biosystems, Waltham, MA). The reaction was performed in a 10-μL volume, containing 5.0 μL of 2 × TaqMan Genotyping Master Mix, 0.5 μL of 20 × genotyping assay mix, and 4.5 μL of DNase-/RNase-free water with 10 ng genomic DNA. Thermal cycling involved an initial enzyme activation at 95°C for 10 min, followed by 40 cycles of denaturation (15 s at 95°C) and annealing/extension (1 min at 60°C). All samples produced valid genotypes (100% call rate), with blinded and replicated quality control samples showing>99% concordance.

### 2.4 Statistical analysis

Statistical analyses were conducted using STATA MP version 18.0 (StataCorp LLC, College Station, TX). Haplotype analysis was performed using SNPStats (https://www.snpstats.net/start.htm). Power calculations were conducted using the online Genetic Association Study (GAS) Power Calculator (https://csg.sph.umich.edu/abecasis/gas_power_calculator/). Categorical variables were compared using the Chi-square test or Fisher’s exact test (for counts <5 in any cell). Continuous variables were analyzed using the Wilcoxon rank-sum test for two groups and the Kruskal–Wallis test for multiple groups. Spearman’s rank correlation assessed associations between SNPs and demographic or clinical variables. All tests were two-sided; *p* < 0.05 was considered statistically significant. Codominant, dominant, recessive, and allelic genetic models were assessed using logistic regression to estimate crude and adjusted odds ratios (ORs and AORs) with 95% confidence intervals (CIs). Adjusted models included selected demographic and clinical covariates: age, gender, ethnicity, and BMI. Duration of diabetes was included only in models where this variable was relevant. The homozygous major allele served as the reference in genotype-based models, while the major allele was the reference in allelic models. Minor and major alleles for each SNP were defined based on allele frequencies observed in our study population. The minor allele was T for rs17470271 (*CYP27A1*) and rs4588 (*GC*), A for rs1074165 (*CYP2R1*), and C for rs7041 (*GC*).

Hardy–Weinberg equilibrium (HWE) was tested in the control group, as well as in alternate groups not genotyped in controls (e.g., rs1074165). Minor allele frequency (MAF) was defined as the frequency of the less common allele and categorized as common (>5%), low-frequency (0.5–5%), or rare (< 0.5%). For comparative purposes, population-level MAF data were obtained from the 1,000 Genomes Project (1000G) (33). Differences in allele frequencies across groups were evaluated using the Chi-square test. Linkage disequilibrium (LD) between SNPs was evaluated using the D′ statistic in SNPstats, and the significance of non-random allelic associations was assessed accordingly. For haplotype analysis, the most frequent haplotype was used as a reference (OR = 1.00).

## 3 Results

### 3.1 Demographic and clinical characteristics

Table 1 summarizes the characteristics of the 170 patients with DKD, 157 DKD-free patients, and 118 healthy controls. The median age was statistically significantly higher in the overall T2DM group and the DKD group compared with controls (*p* < 0.0001), but comparable between DKD and non-DKD participants. While Kazakh ethnicity was statistically significantly more prevalent in the T2DM group compared with controls (*p* < 0.001), and even higher in the DKD group (*p* < 0.001), no significant difference in ethnicity was seen between DKD and non-DKD groups. Sex, geographical origin, marital status, and smoking behavior were comparable across diabetic subgroups.

**Table 1:** Demographic and clinical characteristics of the study groups.

Variable	Diabetes			
	T2DM	DKD group	Non-DKD group	Controls
	*n* = 327	*n* = 170	*n* = 157	*n* = 118
Age, year, median (IQR)	62.7 (56.3–67.6) ¶¶	63.8 (57.3–67.8) †††	61.4 (54.7–67.3)	35.0 (29.0–43.5) ¶¶ †††
Sex, *n* (%)				
Female	177 (54.6)	91 (54.2)	86 (55.1)	52 (61.9)
Male	147 (45.4)	77 (45.8)	70 (44.9)	32 (38.1)
Ethnicity, *n* (%)				
Kazakh	277 (84.7) ¶	148 (87.1) ††	129 (82.2)	77 (65.3) ¶ ††
Non-kazakh	50 (15.3)	22 (12.9)	28 (17.8)	41 (34.7)
Place of living, *n* (%)				
Urban	284 (89.6)	151 (91.5)	133 (87.5)	–
Rural	33 (10.4)	14 (8.5)	19 (12.5)	
Marital status, *n* (%)				
Divorce/divorcee/widow	52 (16.7)	28 (17.2)	24 (16.1)	
Single	11 (3.5)	3 (1.8)	8 (5.4)	–
Married	249 (79.8)	132 (81.0)	117 (78.5)	
Family history of diabetes, *n* (%)				
Yes	165 (52.6)	75 (45.7) *	90 (60.0) *	NA
No	149 (47.4)	89 (54.3)	60 (40.0)	
Diabetes duration, year, median (IQR)	13 (10.1–17)	14 (9.5–19.0)	12.2 (10.4–15.9)	NA
Diabetes duration, year				
<15	194 (59.3)	87 (51.2) **	107 (68.1) **	NA
≥15	133 (40.7)	83 (48.8)	50 (31.9)	
Smoking status, *n* (%)				
Current smoker	37 (11.8)	20 (12.3)	17 (11.3)	–
Former smoker	50 (16.0)	27 (16.6)	23 (15.3)	
Never smoker	226 (72.2)	116 (71.1)	110 (73.4)	
BMI, kg/m^2^, median (IQR)	29.0 (26.1–32.9) ¶	28.6 (25.7–31.3) * †	29.4 (26.4–34.1) *	24.8 (19.4–33.8) ¶ †
BMI, *n* (%)				
Normal (18.5–22.9 kg/m^2^)	17 (5.3) ¶	12 (7.3) ††	5 (3.3)	22 (22.0) ¶ ††
Overweight (23.0–27.4 kg/m^2^)	98 (30.6)	48 (29.1)	50 (32.3)	9 (9.0)
Obese (≥27.5 kg/m^2^)	205 (64.1)	105 (63.6)	100 (64.4)	69 (69.0)
Waist circumference, cm, median (IQR)				
Male	101 (92–108)	102 (94–115)	100 (92–107.5)	–
Female	97 (90–105)	97 (90.5–105)	96 (89–106)	
Waist circumference, *n* (%)				
Non-obese				
- Male (<90 cm)	12 (85.7)	4 (80.0)	8 (88.9)	–
- Female (<80 cm)	2 (14.3)	1 (20.0)	1 (11.1)	
Obese				
- Male (≥90 cm)	135 (43.6)	73 (44.8)	62 (42.2)	–
- Female (≥80 cm)	175 (56.4)	90 (55.2)	85 (57.8)	
Waist-hip ratio, median (IQR)				
Male	0.97 (0.92–1.06)	1 (0.94–1.09)	0.96 (0.92–1.05)	–
Female	0.92 (0.88–0.98)	0.92 (0.88–0.95)	0.92 (0.88–1.01)	
Waist-hip ratio, *n* (%)				
Non-obese				
- Male (<0.90)	10 (37.0)	3 (42.9)	7 (35.0)	–
- Female (<0.85)	17 (63.0)	4 (57.1)	13 (65.0)	
Obese				
- Male (≥0.90)	137 (46.1)	74 (46.0)	63 (46.3)	–
- Female (≥0.85)	160 (53.9)	87 (54.0)	73 (53.7)	
Systolic BP, mmHg, median (IQR)	130 (120–140)	131.5 (120–150) ***	120 (118.5–131.5) ***	–
Diastolic BP, mmHg, median (IQR)	80 (73–86)	80 (76.5–90)	80 (70–85)	–
Arterial hypertension, *n* (%)				
Yes	266 (84.2)	155 (93.4) ***	111 (74.0) ***	–
No	50 (15.8)	11 (6.6)	39 (26.0)	
Myocardial Infarction, *n* (%)				
Yes	43 (13.7)	31 (19.0) **	12 (8.0) **	–
No	270 (86.3)	132 (81.0)	138 (92.0)	
Stroke, *n* (%)				
Yes	44 (14.1)	32 (19.6) **	12 (8.0) **	–
No	269 (85.9)	131 (80.4)	138 (92.0)	
Bronchial asthma, *n* (%)				
Yes	9 (2.8)	2 (1.2)	7 (4.6)	–
No	310 (97.2)	166 (98.8)	144 (95.4)	
Сhronic obstructive pulmonary disease, *n* (%)				
Yes	8 (2.5)	5 (3.0)	3 (2.0)	–
No	311 (97.5)	163 (97.0)	148 (98.0)	
ACE inhibitors/ARBs, *n* (%)				
Yes	141 (45.1)	67 (41.1)	74 (49.3)	–
No	172 (54.9)	96 (58.9)	76 (50.7)	
Statins, *n* (%)				
Yes	68 (21.7)	32 (19.6)	36 (24.0)	–
No	245 (78.3)	131 (80.4)	114 (76.0)	
Antiplatelet agents/anticoagulants, *n* (%)				
Yes	156 (49.8)	78 (47.9)	78 (52.0)	–
No	157 (50.2)	85 (52.1)	72 (48.0)	

Missing data were not evenly distributed across all variables, resulting in minor discrepancies in sample size for specific variables. ACE inhibitors, angiotensin-converting enzyme inhibitors; ARBs, angiotensin II receptor blockers; BMI, body mass index; BP, blood pressure.

Statistical significance between study groups:

DKD vs. non-DKD: *p < 0.05; **p < 0.01; ***p < 0.001.

T2DM vs. controls: ¶*p* < 0.001; ¶¶*p* < 0.0001.

DKD vs. controls: †*p* < 0.01; ††*p* < 0.001; †††*p* < 0.0001.

A family history of diabetes was statistically significantly more common in the non-DKD group than in the DKD group (*p* = 0.01). The median diabetes duration was longer in DKD patients (14.0 years vs. 12.2 years, *p* = 0.08), with a statistically significantly higher proportion of patients having diabetes for >15 years (*p* = 0.002). While BMI was statistically significantly higher in the T2DM group than in controls (*p* < 0.001), and in DKD cases compared with controls (*p* < 0.01), patients with DKD had a statistically significantly lower BMI than non-DKD participants (*p* = 0.03). Compared with the non-DKD group, the DKD group presented with statistically significantly higher median systolic blood pressure (*p* < 0.001), prevalence of hypertension (*p* < 0.001), and cardiovascular/cerebrovascular events, including myocardial infarction (*p* = 0.005) and stroke (*p* = 0.003). Treatment with angiotensin-converting enzyme (ACE) inhibitors/ angiotensin II receptor blockers (ARBs) (*p* = 0.14), statins (*p* = 0.35), and antiplatelet/anticoagulant agents (*p* = 0.46) was similar between DKD and non-DKD groups.

### 3.2 Predictors of diabetic kidney disease

Multivariate logistic regression analysis identified arterial hypertension as the strongest statistically significant independent predictor of DKD (OR = 7.57, 95% CI: 3.32–17.27, *p* < 0.001), followed by stroke (OR = 2.48, 95% CI: 1.11–5.54, *p* = 0.03). Specifically, a higher BMI was associated with a statistically significant inverse risk of DKD (OR = 0.96, 95% CI: 0.92–0.99, *p* = 0.02), as was the use of ACE inhibitors or ARBs (OR = 0.50, 95% CI: 0.29–0.86, *p* = 0.01) (Table 2).

**Table 2:** Multivariable logistic regression analysis of the demographic and clinical risk factors associated with diabetic kidney disease.

Variable	Model 1		Model 2		Model 3	
	*p*-value	OR (95% CI)	*p*-value	OR (95% CI)	*p*-value	OR (95% CI)
Age	**0.03**	**1.03 (1.004–1.06)**	0.12	1.02 (0.99–1.05)	0.27	1.02 (0.99–1.05)
Gender	0.75	0.93 (0.59–1.46)	0.77	0.93 (0.59–1.48)	0.80	0.94 (0.56–1.56)
Ethnicity	0.17	1.56 (0.83–2.95)	0.16	1.59 (0.84–3.03)	0.05	2.04 (0.99–4.22)
BMI	–	–	0.06	0.97 (0.94–1.001)	**0.02**	**0.96 (0.92–0.99)**
Diabetes duration	–	–	0.07	1.04 (0.99–1.09)	0.20	1.03 (0.98–1.08)
Arterial hypertension	–	–	–	–	**< 0.001**	**7.57 (3.32–17.27)**
Myocardial Infarction	–	–	–	–	0.07	2.17 (0.95–4.97)
Stroke	–	–	–	–	**0.03**	**2.48 (1.11–5.54)**
ACE inhibitors/ARBs	–	–	–	–	**0.01**	**0.50 (0.29–0.86)**
Statins	–	–	–	–	0.18	0.65 (0.35–1.22)
Antiplatelet agents/anticoagulants	–	–	–	–	0.41	0.80 (0.47–1.36)

ACE inhibitors, angiotensin-converting enzyme inhibitors; ARBs, angiotensin II receptor blockers; BMI, body mass index. Boldface indicates statistically significant differences (*p* < 0.05).

### 3.3 Association of vitamin D metabolism pathway gene variants with diabetic kidney disease risk

**Figure 1:**
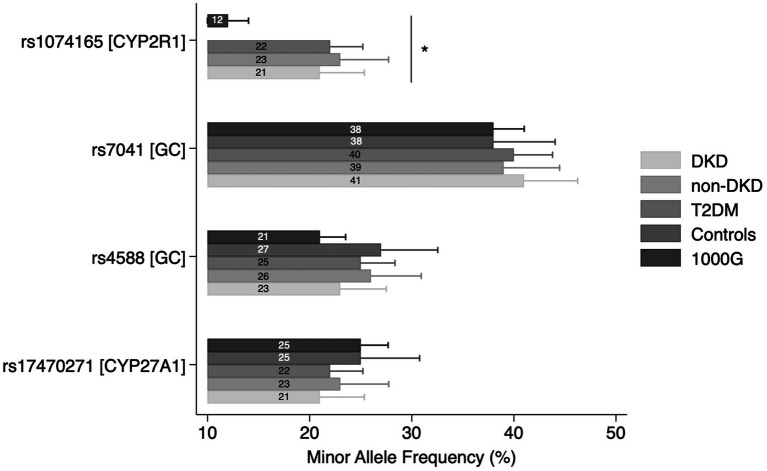
Minor allele frequencies (MAF) comparison across study groups. Bars represent MAF values with 95% confidence intervals. *p < 0.0001.

No significant departures from HWE were noted for rs17470271, rs4588, and rs7041 in controls. No deviations were also seen for rs1074165, assessed in DKD, non-DKD, and T2DM groups. The MAF comparisons across study groups and the general population are presented in Supplementary Table S1 and Figure 1. A statistically significant difference in the MAF of rs1074165 was noted between all groups (DKD, non-DKD, T2DM, and general population) (*p* < 0.0001). In contrast, the MAF of rs17470271, rs4588, and rs7041 were not significantly different between the tested groups.

**Figure 2:**
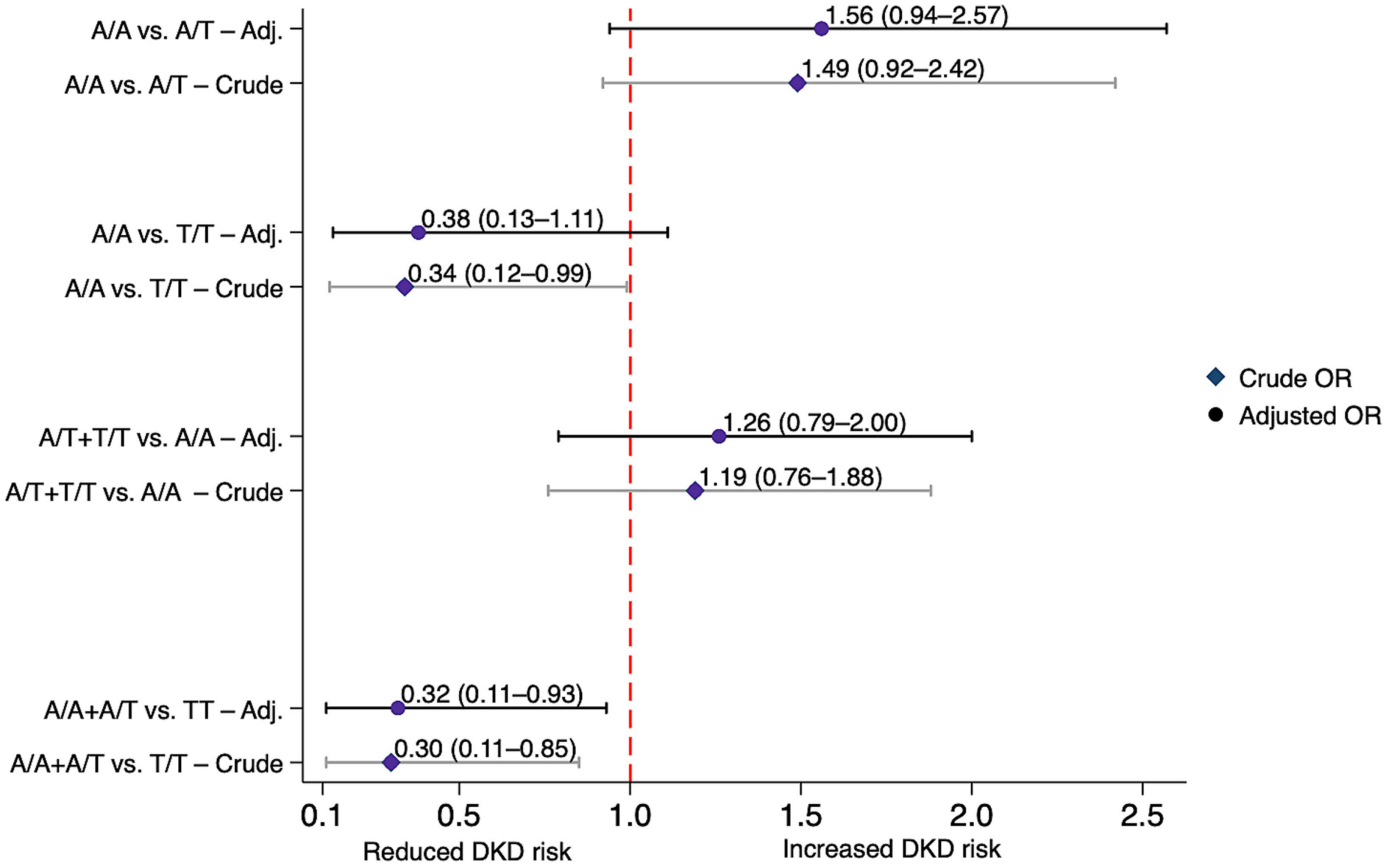
Association between the *CYP27A1* rs17470271 variant and diabetic kidney disease risk under various genetic models in individuals with and without diabetic kidney disease. Crude and adjusted (Adj.) odds ratios (ORs) with 95% confidence intervals (CIs) for each model. Adjusted ORs were corrected for age, gender, ethnicity, body mass index (BMI) and diabetes duration. DKD, diabetic kidney disease.

Table 3 summarizes the association of gene polymorphisms with DKD risk in DKD and non-DKD groups. For *CYP27A1* rs17470271, T/T genotype carriers had a statistically significant lower risk of DKD compared with A/A carriers under both the codominant (OR = 0.34, 95% CI: 0.12–0.99, *p* = 0.04) and recessive (OR = 0.30, 95% CI: 0.11–0.85, *p* = 0.024) models. The association remained statistically significant in the recessive model after adjustment (AOR = 0.32, 95% CI: 0.11–0.93, *p* = 0.036). No similar associations were seen in the dominant or allelic models. Figure 2 visualizes these associations.

**Table 3:** Association between vitamin D metabolism pathway gene polymorphisms and diabetic kidney disease (DKD) risk: comparison between individuals with and without DKD.

SNP *[Gene]*	Genetic model	DKD group, *n* (%)	Non-DKD group, *n* (%)	C-OR (95% CI)	*p-*value	A-OR* (95% CI)	*p-*value
rs17470271 *[CYP27A1]*	Codominant						
	A/A	101 (60.1)	97 (64.2)	Ref.	–	Ref.	–
	A/T	62 (36.9)	40 (26.5)	1.49 (0.92–2.42)	0.11	1.56 (0.94–2.57)	0.08
	T/T	5 (3.0)	14 (9.3)	**0.34 (0.12–0.99)**	**0.04**	0.38 (0.13–1.11)	0.08
	Dominant						
	A/A	101 (60.1)	97 (64.2)	Ref.	0.45	Ref.	0.34
	A/T + T/T	67 (39.9)	54 (35.8)	1.19 (0.76–1.88)		1.26 (0.79–2.00)	
	Recessive						
	A/A + A/T	163 (97.0)	137 (90.7)	Ref.	**0.024**	Ref.	**0.036**
	T/T	5 (3.0)	14 (9.3)	**0.30 (0.11–0.85)**		**0.32 (0.11–0.93)**	
	Allelic						
	A	264 (78.6)	234 (77.5)	Ref.	0.75	Ref.	0.93
	T	72 (21.4)	68 (22.5)	0.94 (0.66–1.36)		0.98 (0.68–1.43)	
rs4588 *[GC]*	Codominant						
	G/G	98 (58.3)	85 (55.9)	Ref.	–	Ref.	–
	G/T	63 (37.5)	54 (35.5)	1.01 (0.64–1.61)	0.96	1.05 (0.64–1.72)	0.84
	T/T	7 (4.2)	13 (8.6)	0.47 (0.18–1.22)	0.12	**0.28 (0.09–0.85)**	**0.025**
	Dominant						
	G/G	98 (58.3)	85 (55.9)	Ref.	0.66	Ref.	0.63
	G/T + T/T	70 (41.7)	67 (44.1)	0.91 (0.58–1.41)		0.89 (0.56–1.43)	
	Recessive						
	G/G + G/T	161 (95.8)	139 (91.4)	Ref.	0.11	Ref.	**0.029**
	T/T	7 (4.2)	13 (8.6)	0.46 (0.18–1.20)		**0.30 (0.10–0.88)**	
	Allelic						
	G	259 (77.1)	224 (73.7)	Ref.	0.32	Ref.	0.20
	T	77 (22.9)	80 (26.3)	0.83 (0.58–1.19)		0.78 (0.53–1.14)	
rs7041 *[GC]*	Codominant						
	A/A	56 (33.3)	58 (38.2)	Ref.	–	Ref.	–
	A/C	87 (51.8)	68 (44.7)	1.33 (0.82–2.15)	0.26	1.53 (0.91–2.56)	0.11
	C/C	25 (14.9)	26 (17.1)	0.99 (0.52–1.93)	0.99	1.36 (0.64–2.85)	0.42
	Dominant						
	A/A	56 (33.3)	58 (38.2)	Ref.	0.37	Ref.	0.11
	A/C + C/C	112 (66.7)	94 (61.8)	1.23 (0.78–1.95)		1.48 (0.91–2.41)	
	Recessive						
	A/A + A/C	143 (85.1)	126 (82.9)	Ref.	0.59	Ref.	0.85
	C/C	25 (14.9)	26 (17.1)	0.85 (0.47–1.54)		1.06 (0.56–2.00)	
	Allelic						
	A	199 (59.2)	184 (60.5)	Ref.	0.74	Ref.	0.23
	C	137 (40.8)	120 (39.5)	1.06 (0.77–1.45)		1.23 (0.88–1.73)	
rs1074165 *[CYP2R1]*	Codominant						
	G/G	105 (62.5)	87 (57.2)	Ref.	–	Ref.	–
	G/A	55 (32.7)	61 (40.2)	0.75 (0.47–1.19)	0.22	0.63 (0.39–1.04)	0.07
	A/A	8 (4.8)	4 (2.6)	1.66 (0.48–5.69)	0.42	1.45 (0.39–5.36)	0.58
	Dominant						
	G/G	105 (62.5)	87 (57.2)	Ref.	0.34	Ref.	0.11
	G/A + A/A	63 (37.5)	65 (42.8)	0.80 (0.51–1.26)		0.68 (0.42–1.09)	
	Recessive						
	G/G + G/A	160 (95.2)	148 (97.4)	Ref.	0.32	Ref.	0.44
	A/A	8 (4.8)	4 (2.6)	1.85 (0.55–6.27)		1.67 (0.46–6.14)	
	Allelic						
	G	265 (78.9)	235 (77.3)	Ref.	0.62	Ref.	0.25
	A	71 (21.1)	69 (22.7)	0.91 (0.62–1.34)		0.78 (0.52–1.19)	

*Adjusted by age, gender, ethnicity, body mass index (BMI), and duration of diabetes. A-OR, adjusted odds ratio; CI, confidence interval; C-OR, crude odds ratio. Boldface indicates statistically significant differences (*p* < 0.05).

**Figure 3:**
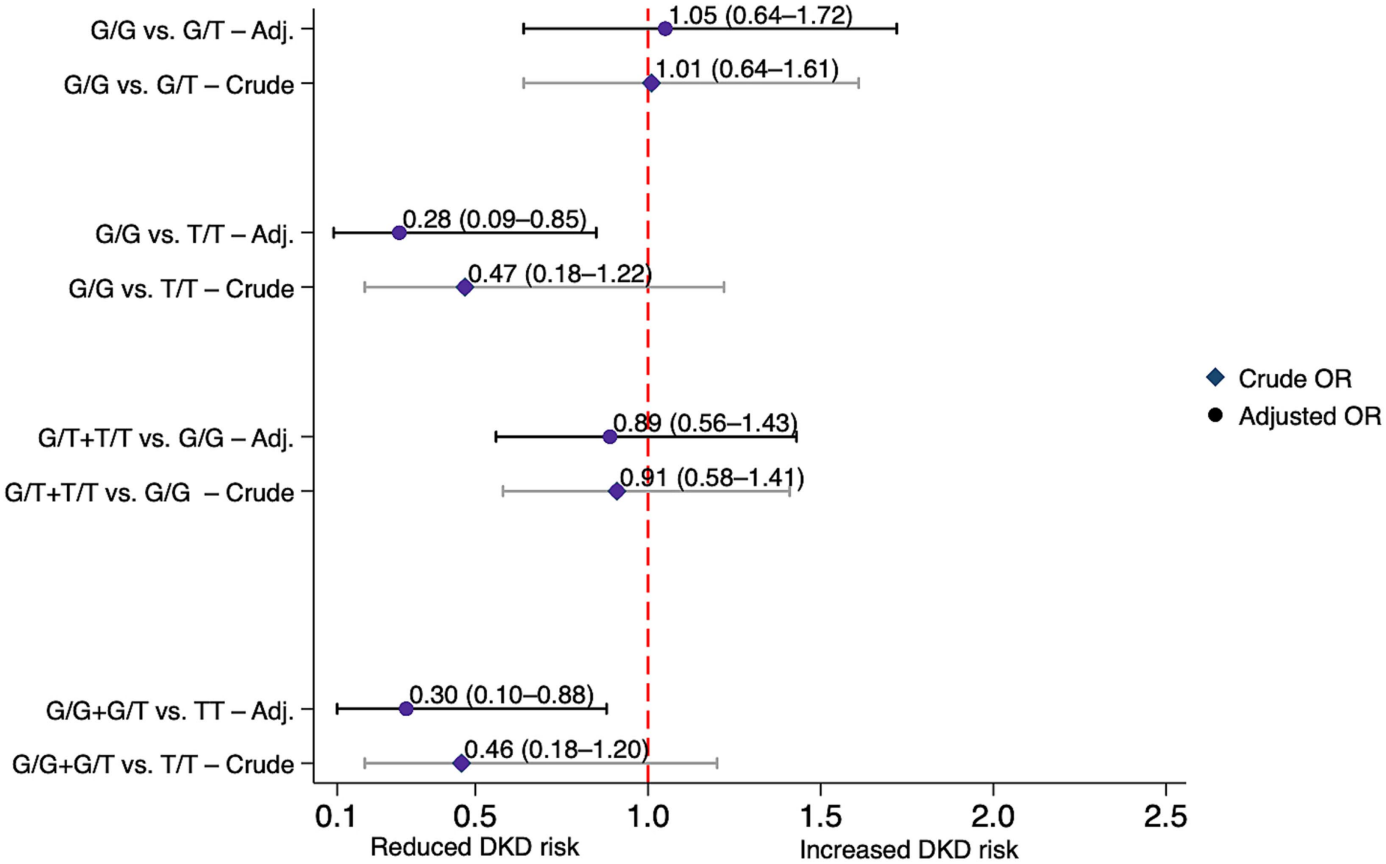
Association between the *GC* rs4588 variant and diabetic kidney disease risk under various genetic models in individuals with and without diabetic kidney disease. Crude and adjusted (Adj.) odds ratios (ORs) with 95% confidence intervals (CIs) for each model. Adjusted ORs were corrected for age, gender, ethnicity, body mass index (BMI) and diabetes duration. DKD, diabetic kidney disease.

Compared with *GC* rs4588 G/G genotype carriers, individuals with the T/T genotype exhibited a statistically significant reduction in DKD risk under the codominant model after adjustment (AOR = 0.28, 95% CI: 0.09–0.85, *p* = 0.025), and this finding was confirmed by the recessive model (AOR = 0.30, 95% CI: 0.10–0.88, *p* = 0.029) (Table 3). Figure 3 illustrates these associations. On the other hand, both *GC* rs7041 and *CYP2R1* rs1074165 were not significantly associated with altered risk of DKD under any genetic models, possibly due to limited power, modest effect sizes, or involvement in pathways less directly related to DKD.

Supplementary Tables S2, S3 present associations between vitamin D metabolism gene polymorphisms and the risks of DKD and T2DM, respectively, compared with conditionally healthy controls. When comparing participants with DKD to conditionally healthy controls, the codominant model revealed that rs4588 T/T genotype carriers had a statistically significantly reduced risk of DKD compared with individuals carrying the G/G genotype (AOR = 0.07, 95% CI: 0.006–0.98, *p* = 0.048), with a similar, albeit borderline, significance in the recessive model (AOR = 0.10, 95% CI: 0.009–1.06, *p* = 0.055) (Supplementary Table S2). In contrast, *GC* rs7041 and *CYP27A1* rs17470271 showed no similar associations under any genetic model in either analysis.

*Post hoc* power analysis, based on comparisons between DKD and the non-DKD group, indicated >80% power to detect the observed associations of *GC* rs4588 and *CYP27A1* rs17470271 with DKD under recessive genetic models, which exhibited the most robust effect sizes.

### 3.4 Gene-disease interaction of *CYP27A1* and *GC* polymorphisms with diabetic kidney disease

No significant differences were observed in demographic, clinical, or pharmacological factors across *CYP27A1* rs17470271 genotypes in the DKD or non-DKD groups under dominant, recessive, and genotypic models, except for the statistically significant higher prevalence of stroke among A/A carriers in the non-DKD group under the dominant model (*p* = 0.038) (Supplementary Table S4). This confirms the independence of the association between *CYP27A1* rs17470271 T/T genotype and DKD risk from the main confounding factors. Stratified analysis of *GC* rs4588 identified ethnicity as a significant effect modifier in the gene-disease interaction (Supplementary Table S5). Within the DKD group, statistically significant associations between genotype and ethnicity were seen under the genotypic (*p* = 0.001) and recessive (*p* < 0.001) genetic models, suggesting ethnicity-dependent variation in rs4588 genotype distribution. These findings imply that the protective effect of the rs4588 T/T genotype may vary between ethnic subgroups, potentially due to population-specific allele frequencies or gene–environment interactions.

### 3.5 Correlation studies

The correlation between *CYP27A1* rs17470271 and *GC* rs4588 polymorphisms and the analyzed demographic/clinical characteristics is shown in Supplementary Table S6. Among the non-DKD group, the *CYP27A1* rs17470271 genotype showed weak, statistically significant inverse correlations with marital status (*ρ* = − 0.19, *p* = 0.03) and stroke (*ρ* = − 0.16, *p* = 0.048). Conversely, *GC* rs4588 was inversely correlated with ethnicity in the total population (*ρ* = −0.17, *p* = 0.003) and in the non-DKD group (*ρ* = −0.20, *p* = 0.02), while showing a modest, statistically significant positive correlation with stroke in the non-DKD group (*ρ* = 0.20, *p* = 0.01).

### 3.6 Haplotype-based analysis of diabetic kidney disease susceptibility within the *GC* gene

The association of the two-locus *GC* rs4588-rs7041 haplotypes and DKD susceptibility is presented in Table 4. LD analysis showed moderate linkage between rs4588 and rs7041 (D′ = 0.68, *p* < 0.0001), indicating non-random allelic co-inheritance. The *G-C* haplotype was associated with an increased DKD risk (crude OR = 1.52, 95% CI: 1.02–2.28, *p* = 0.043), which remained statistically significant after adjustment for age, gender, and ethnicity (AOR = 3.15, 95% CI: 1.21–8.18, *p* = 0.02). No significant multi-locus allele combinations were identified when comparing individuals with and without DKD (Supplementary Table S7).

**Table 4:** Haplotype-based analysis of *GC* variants with susceptibility to diabetic kidney disease (DKD): comparison between individuals with DKD and conditionally healthy controls.

Block	rs4588 *[GC]*	rs7041 *[GC]*	DKD, frequency	Controls, frequency	C-OR (95% CI)	*p*-value	A-OR* (95% CI)	*p*-value	D′	LD *p*-value
1	G	A	0.36	0.42	Ref.	–	Ref.	–	–	–
2	G	C	0.41	0.31	**1.52 (1.02–2.28)**	**0.043**	**3.15 (1.21–8.18)**	**0.02**	**0.68**	**< 0.0001**
3	T	A	0.23	0.20	1.35 (0.84–2.17)	0.22	2.16 (0.69–6.71)	0.19	–	–
4	T	C	0	0.07	–	–	–	–	–	–

*Adjusted by age, gender, and ethnicity. A-OR, adjusted odds ratio; CI, confidence interval; C-OR, crude odds ratio; DKD, diabetic kidney disease. Boldface indicates statistically significant differences (*p* < 0.05).

## 4 Discussion

### 4.1 Key findings and population-specific context

This study investigated associations between common polymorphisms in key vitamin D metabolism genes and DKD susceptibility in T2DM patients from Kazakhstan. To our knowledge, this is the first investigation of *CYP27A1* rs17470271 and *CYP2R1* rs1074165 in relation to DKD. Significant associations were observed between the *CYP27A1* rs17470271 T/T genotype and a reduced risk of DKD under both recessive and codominant genetic models. Similarly, carriers of the *GC* rs4588 T/T genotype were linked with a reduced risk of DKD. Beyond single-locus effects, our haplotype analysis revealed that the *rs4588G-rs7041C* haplotype of the *GC* gene was associated with an increased risk of DKD compared with healthy controls, suggesting that combinatorial genetic effects may affect susceptibility rather than DKD progression in diabetic individuals.

These findings were derived from participants representing Kazakhstan’s multi-ethnic population (26), whose genetic diversity may contribute to unique patterns of disease susceptibility not captured in more homogeneous cohorts. To better contextualize our results, we examined allele frequency distributions in our study population relative to the 1000G reference. Among the studied SNPs, only *CYP2R1* rs1074165 exhibited a statistically significant difference in MAF compared with the 1000G reference population, potentially reflecting population-specific variation in allele distribution. Based on available databases, there’s no current evidence that this SNP resides within a known regulatory element. Notably, *CYP2R1* rs1074165 was not genotyped in the healthy control group, and comparisons were therefore limited to the diabetic subgroups and external reference data. The remaining SNPs (*CYP27A1* rs17470271, *GC* rs4588 and rs7041) demonstrated similar frequencies to global reference values, indicating broadly conserved patterns across populations. Based on established thresholds, all four variants were classified as common polymorphisms (MAF > 5%) both in our cohort and in the reference data, underscoring their relevance for population-level association analyses.

We demonstrated that the *GC* rs4588 variant is significantly associated with DKD compared with both DKD-free patients and healthy controls, suggesting a potential role in both diabetes-to-DKD progression and kidney dysfunction. On the other hand, *CYP27A1* rs17470271 was not significantly associated when DKD cases were compared with healthy controls, indicating potential involvement in diabetes-to-DKD progression rather than the risk of general kidney disease. This could also result from reduced statistical power or population-specific factors, including environmental exposures or genetic heterogeneity affecting the *CYP27A1* signal.

The higher prevalence of non-Kazakh ethnicity among *GC* rs4588 T/T genotype carriers noted within the DKD group warrants caution in interpreting the results due to the limited sample size and the discrepancy with the correlation analysis. Ethnicity may act as a confounding factor, potentially influencing both genotype distribution and disease risk through underlying population structure. These findings underscore the importance of considering ancestry in genetic association studies and highlight the need for replication in larger, multi-ethnic cohorts to determine the generalizability of *GC*-related associations with DKD.

### 4.2 Interpretation in relation to previous studies

Previous studies examining the association of *GC* rs7041 and rs4588 with T2DM susceptibility have reported inconsistent findings across different populations. In a Bangladeshi cohort, both *GC* rs7041 G/G and *GC* rs4588 A/A genotypes reportedly increased the risk of T2DM (22). A Chinese study confirmed the risk association for *GC* rs7041 G/G but found *GC* rs4588 to be protective under a dominant model (34), underscoring population-level heterogeneity within Asia. Among Saudi cohorts, *GC* rs4588 A allele carriers were more common among T2DM patients, and the A/A genotype was associated with albuminuria, elevated DBP, and poor glycemic control (35). Conversely, European studies from French (36) and Polish (37) populations showed no significant associations between *GC* variants and T2DM. The lack of significant association between *GC* rs7041 or rs4588 and T2DM in our study may be attributed to population genetics differences, environmental exposures, sample sizes, or study design.

Although Fawzy et al. (35) reported findings that contrast with our observation of a protective effect for the *GC* rs4588 T/T genotype against DKD, these discrepancies may be due to differences in genetic background, DKD definitions (albuminuria vs. clinically defined DKD), or gene–environment interactions. The interpretation is further complicated by mixed biological and clinical evidence. On the one hand, it is likely that the *GC* rs4588 AA genotype is associated with lower circulating 25(OH)D concentrations (38, 39), which in turn are linked to an increased risk of DKD (40). On the other hand, Gc2 homozygotes showed lower incremental glucose during the OGTT (41), suggesting favorable glucose metabolism and thus reduced DKD risk (42). Since metabolic syndrome constitutes an independent predictor of DKD (43), the findings of Zhao et al. (21) support our results from a complementary perspective. They reported that *GC* rs4588 CA/AA carriers had lower metabolic syndrome risk than CC carriers, potentially due to favorable lipid profiles. Overall, these opposing observations highlight the complexity of *GC* gene effects on DKD pathophysiology and underscore the need for further research, particularly studies that integrate genetic, environmental, and metabolic factors across diverse populations.

While earlier studies focused on *GC* variants, this is the first study to examine the association between *CYP27A1* rs17470271 and DKD risk, underscoring the novelty of our findings. The *CYP27A1* rs17470271 variant was reported in only a few studies, including a study from China, which linked it with leukopenia in patients with pulmonary tuberculosis (44). In support of its relevance, transcription factor loss-of-function (TF-LOF) enrichment analysis from the GEO 2022 dataset (Enrichr) identified *CYP27A1* among genes upregulated in estrogen-related receptor alpha (ESRRA)-deficient mouse kidney tissue (GSE16623; adjusted *p* = 0.04) (45–47), suggesting potential ESRRA-mediated regulatory influence. Insofar as it controls proximal tubule metabolism, which is presumably protective of kidney injury (48), reduced ESRRA expression was linked to early mitochondrial loss in proximal tubular epithelial cells, a key event in DKD onset (49). While these findings suggest that *CYP27A1* expression may be influenced by ESRRA-related metabolic pathways in the diabetic kidney, they remain hypothesis-generating and do not imply a causal relationship.

### 4.3 Biological relevance and hypothesized mechanisms

Vitamin D activity is mediated by its metabolites, which are regulated by proteins involved in transport and metabolism. The *GC* gene encodes the DBP, which binds 25(OH)D in circulation and enables its uptake into proximal tubular cells via megalin–cubilin–mediated endocytosis, which then dissociates and converts to the 1,25(OH)_2_D active form (50). *CYP27A1* encodes a mitochondrial enzyme that catalyzes the 25-hydroxylation of vitamin D_3_ (12) and plays a role in cholesterol metabolism and bile acid synthesis (51). Dysfunction in the latter pathway may lead to lipid accumulation (52), in turn contributing to DKD progression (53). It is plausible that genetic variants in *GC* and *CYP27A1* genes influence DKD susceptibility by altering systemic and renal vitamin D dynamics. Notably, *GC* rs7041 and rs4588 polymorphisms are associated with lower 25(OH)D levels, due to altered structure of DBP, glycosylation, and turnover (54), and with changes in binding affinity for vitamin D metabolites (55). Moreover, *GC* variants were linked to lipid profiles, suggesting broader metabolic implications (56). Unlike *GC*, the role of *CYP27A1* in regulating circulating 25(OH)D remains not completely defined (57).

These findings raise the possibility that *GC* polymorphisms may enhance vitamin D bioavailability or renal delivery, thus promoting VDR-mediated protective effects in diabetic kidneys. Despite their link to reduced total 25(OH)D, *GC* variants may alter DBP binding affinity, increasing the bioavailable fraction, and may also influence lipid metabolism through effects on hepatic lipid regulation, insulin sensitivity, and inflammation. Similarly, *CYP27A1* variants may hypothetically support the efficient conversion of precursors to 25(OH)D under hyperglycemic conditions, sustaining intrarenal calcitriol synthesis. Separately, given *CYP27A1*’s established role in cholesterol homeostasis, protective variants may also mitigate lipid accumulation and associated renal injury. These proposed mechanisms require functional validation, including studies in diabetic *in vivo* models such as *GC* and *CYP27A1* knockout mice, and may be further supported by integrative renal omics approaches (e.g., transcriptomics, proteomics, metabolomics) to clarify their role in the pathogenesis of DKD.

## 5 Limitations and future directions

This study has several important limitations that must be acknowledged. First, the exploratory design of the study and multiple comparisons warrant cautious interpretation and appropriate correction for multiple testing. Second, as it is an observational study, it thereby necessitates care in extrapolating cause-effect relationships, as the identified associations may be affected by measured and unmeasured confounders. Third, while we controlled for key environmental and lifestyle factors that potentially affect vitamin D metabolism, the possibility of the contribution of other modifiable and non-modifiable factors to the effects seen cannot be dismissed. Fourth, we acknowledge that the relatively small sample size may have reduced the statistical power, particularly in genotype-stratified analyses, supporting the need for larger study cohorts. Fifth, the absence of serum vitamin D measurements raises speculation on the functional impact of the studied variants on vitamin D status. Lastly, the study subjects were of Kazakhstani origin, thus questioning the universality of these findings, and recommending replication in larger, multiethnic cohorts. Despite these shortcomings, our result establishes a strong contribution of vitamin D-related pathways in the pathogenesis of DKD, which will be instrumental in future precision medicine approaches.

## 6 Conclusion

This study identified significant associations between polymorphisms in vitamin D metabolic pathway genes and the risk of DKD among individuals with T2DM from the Kazakhstani population. These findings support the proposed role of vitamin D pathway genes in DKD susceptibility and may reflect population-specific genetic and environmental factors influencing the observed associations. While the study has certain limitations that may constrain its generalizability and mechanistic interpretation, the findings contribute novel insight into the genetic architecture of DKD. Future large-scale, multiethnic studies with functional validation are needed to confirm these associations and translate them into improved risk prediction and precision prevention strategies.

## Data Availability

The original contributions presented in the study are publicly available. This data can be found here: https://doi.org/10.17632/9ts2bjjrbs.1.
