# Supplementary material for: Genetic variants in the vitamin D pathway genes are predictors of the risk of diabetic kidney disease in Central Asian Kazakhstani cohort with type 2 diabetes
Source: Front Med (Lausanne). 2025 Jul 10;12:1630725. doi: 10.3389/fmed.2025.1630725 (PMC12286948; doi:10.3389/fmed.2025.1630725)
Supplement: Supplementary file 1 [file Table_1.docx]

Supplementary Materials

**Table S1.** Minor allele frequencies (MAF) comparison across study groups.

|  | **MAF in DKD** | **MAF in non-DKD** | **MAF in T2DM** | **MAF in Controls** | **MAF in general population, 1000G** | ***p*-value** |
| --- | --- | --- | --- | --- | --- | --- |
| rs17470271 | 0.21 | 0.23 | 0.22 | 0.25 | 0.25 | 0.53 |
| rs4588 | 0.23 | 0.26 | 0.25 | 0.27 | 0.21 | 0.15 |
| rs7041 | 0.41 | 0.39 | 0.40 | 0.38 | 0.38 | 0.83 |
| rs1074165 | **0.21** | **0.23** | **0.22** | **–** | **0.12** | **<0.0001** |

MAF, minor allele frequencies. Boldface indicates statistically significant differences (*p* < 0.05).

**Table S2.** Genetic association analysis of individuals with diabetic kidney disease versus conditionally healthy controls.

| **SNP *[Gene]*** | **Genetic model** | **DKD, n (%)** | **Controls, n (%)** | **C-OR (95% CI)** | ***p-*value** | **A-OR* (95% CI)** | ***p-*value** |
| --- | --- | --- | --- | --- | --- | --- | --- |
| rs17470271  *[CYP27A1]* | Codominant | | |  |  |  |  |
|  | A/A | 101 (60.1) | 57 (55.4) | Ref. | – | Ref. | – |
|  | A/T | 62 (36.9) | 40 (38.8) | 0.87 (0.52 – 1.46) | 0.61 | 0.97 (0.34 – 2.78) | 0.95 |
|  | T/T | 5 (3.0) | 6 (5.8) | 0.47 (0.14 – 1.61) | 0.23 | 1.84 (0.20 – 17.0) | 0.54 |
|  | Dominant | | |  |  |  |  |
|  | A/A | 101 (60.1) | 57 (55.3) | Ref. | 0.44 | Ref. | 0.95 |
|  | A/T+T/T | 67 (39.9) | 46 (44.7) | 0.82 (0.50 – 1.35) |  | 1.03 (0.38 – 2.85) |  |
|  | Recessive |  |  |  |  |  |  |
|  | A/A+A/T | 163 (97.0) | 97 (94.2) | Ref. | 0.26 | Ref. | 0.58 |
|  | T/T | 5 (3.0) | 6 (5.8) | 0.52 (0.15 – 1.67) |  | 1.82 (0.22 – 15.0) |  |
|  | Allelic |  |  |  |  |  |  |
|  | A | 264 (78.6) | 154 (74.8) | Ref. | 0.29 | Ref. | 0.78 |
|  | T | 72 (21.4) | 52 (25.2) | 0.79 (0.52– 1.22) |  | 1.12 (0.49 – 2.56) |  |
| rs4588 *[GC]* | Codominant | | |  | |  |  |
|  | G/G | 98 (58.3) | 63 (53.9) | Ref. | – | Ref. | – |
|  | G/T | 63 (37.5) | 44 (37.6) | 0.92 (0.56 – 1.52) | 0.74 | 0.76 (0.27 – 2.10) | 0.60 |
|  | T/T | 7 (4.2) | 10 (8.5) | 0.45 (0.16 – 1.24) | 0.12 | **0.07 (0.006 – 0.98)** | **0.048** |
|  | Dominant | | |  | |  |  |
|  | G/G | 98 (58.3) | 63 (53.9) | Ref. | 0.45 | Ref. | 0.31 |
|  | G/T+T/T | 70 (41.7) | 54 (46.1) | 0.83 (0.52 – 1.34) |  | 0.60 (0.22 – 1.62) |  |
|  | Recessive | | |  | |  |  |
|  | G/G+G/T | 161 (95.8) | 107 (91.5) | Ref. | 0.13 | Ref. | **0.055** |
|  | T/T | 7 (4.2) | 10 (8.5) | 0.47 (0.17 – 1.26) |  | **0.10 (0.009 – 1.06)** |  |
|  | Allelic | | |  | |  |  |
|  | G | 259 (77.1) | 170 (72.6) | Ref. | 0.23 | Ref. | 0.11 |
|  | T | 77 (22.9) | 64 (27.4) | 0.79 (0.53 – 1.16) |  | 0.51 (0.22 – 1.18) |  |
| rs7041  *[GC]* | Codominant | | |  | |  |  |
|  | A/A | 56 (33.3) | 45 (38.1) | Ref. | – | Ref. | – |
|  | A/C | 87 (51.8) | 56 (47.5) | 1.25 (0.75 – 2.09) | 0.40 | 1.26 (0.41 – 3.90) | 0.69 |
|  | C/C | 25 (14.9) | 17 (14.4) | 1.18 (0.57 – 2.45) | 0.65 | 1.57 (0.24 – 10.13) | 0.64 |
|  | Dominant | | |  | |  |  |
|  | A/A | 56 (33.3) | 45 (38.1) | Ref. | 0.40 | Ref. | 0.62 |
|  | A/C+C/C | 112 (66.7) | 73 (61.9) | 1.23 (0.75 – 2.01) |  | 1.32 (0.45 – 3.84) |  |
|  | Recessive | | |  | |  |  |
|  | A/A+A/C | 143 (85.1) | 101 (85.6) | Ref. | 0.91 | Ref. | 0.46 |
|  | C/C | 25 (14.9) | 17 (14.4) | 1.04 (0.53 – 2.02) |  | 1.61 (0.45 – 5.79) |  |
|  | Allelic | | |  | |  |  |
|  | A | 199 (59.2) | 146 (61.9) | Ref. | 0.52 | Ref. | 0.45 |
|  | C | 137 (40.8) | 90 (38.1) | 1.12 (0.79 – 1.59) |  | 1.32 (0.64 – 2.71) |  |

*** Adjusted by age, gender, ethnicity and body mass index (BMI). A-OR, adjusted odds ratio; CI, confidence interval; C-OR, crude odds ratio. Boldface indicates statistically significant differences (*p* < 0.05) as well as borderline significance.

**Table S3.** Genetic association analysis of individuals with type 2 diabetes mellitus versus conditionally healthy controls.

| **SNP *[Gene]*** | **Genetic model** | **T2DM, n (%)** | **Controls, n (%)** | **C-OR (95% CI)** | ***p-*value** | **A-OR* (95% CI)** | ***p-*value** |
| --- | --- | --- | --- | --- | --- | --- | --- |
| rs17470271  *[CYP27A1]* | Codominant | | |  |  |  |  |
|  | A/A | 198 (62.0) | 57 (55.4) | Ref. | – | Ref. | – |
|  | A/T | 102 (32.0) | 40 (38.8) | 0.73 (0.46 – 1.17) | 0.20 | 0.71 (0.30 – 1.71) | 0.45 |
|  | T/T | 19 (6.0) | 6 (5.8) | 0.91 (0.35 – 2.39) | 0.85 | 1.43 (0.24 – 8.42) | 0.69 |
|  | Dominant | | |  |  |  |  |
|  | A/A | 198 (62.1) | 57 (55.3) | Ref. | 0.23 | Ref. | 0.59 |
|  | A/T+T/T | 121 (37.9) | 46 (44.7) | 0.78 (0.48 – 1.19) |  | 0.80 (0.34 – 1.84) |  |
|  | Recessive |  |  |  |  |  |  |
|  | A/A+A/T | 300 (94.0) | 97 (94.2) | Ref. | 0.96 | Ref. | 0.59 |
|  | T/T | 19 (6.0) | 6 (5.8) | 1.02 (0.40 – 2.64) |  | 1.57 (0.30 – 8.24) |  |
|  | Allelic |  |  |  |  |  |  |
|  | A | 498 (78.1) | 154 (74.8) | Ref. | 0.33 | Ref. | 0.85 |
|  | T | 140 (21.9) | 52 (25.2) | 0.84 (0.59– 1.20) |  | 0.94 (0.49– 1.80) |  |
| rs4588 *[GC]* | Codominant | | |  |  |  |  |
|  | G/G | 183 (57.2) | 63 (53.9) | Ref. | – | Ref. | – |
|  | G/T | 117 (36.6) | 44 (37.6) | 0.92 (0.58 – 1.44) | 0.70 | 1.09 (0.47 – 2.57) | 0.84 |
|  | T/T | 20 (6.2) | 10 (8.5) | 0.69 (0.31 – 1.55) | 0.37 | 0.38 (0.07 – 2.22) | 0.28 |
|  | Dominant | | |  |  |  |  |
|  | G/G | 183 (57.2) | 63 (53.9) | Ref. | 0.53 | Ref. |  |
|  | G/T+T/T | 137 (42.8) | 54 (46.1) | 0.87 (0.57 – 1.34) |  | 0.96 (0.42 – 2.16) | 0.92 |
|  | Recessive | | |  |  |  |  |
|  | G/G+G/T | 300 (93.8) | 107 (91.5) | Ref. | 0.40 | Ref. | 0.23 |
|  | T/T | 20 (6.2) | 10 (8.5) | 0.71 (0.32 – 1.57) |  | 0.37 (0.07 – 1.87) |  |
|  | Allelic | | |  |  |  |  |
|  | G | 483 (75.5) | 170 (72.6) | Ref. | 0.40 | Ref. | 0.58 |
|  | T | 157 (24.5) | 64 (27.4) | 0.87 (0.62 – 1.21) |  | 0.83 (0.42 – 1.61) |  |
| rs7041  *[GC]* | Codominant | | |  |  |  |  |
|  | A/A | 114 (35.6) | 45 (38.1) | Ref. | – | Ref. | – |
|  | A/C | 155 (48.4) | 56 (47.5) | 1.09 (0.69 – 1.73) | 0.70 | 0.82 (0.33 – 2.05) | 0.68 |
|  | C/C | 51 (16.0) | 17 (14.4) | 1.18 (0.62 – 2.26) | 0.61 | 0.65 (0.15 – 2.76) | 0.56 |
|  | Dominant | | |  |  |  |  |
|  | A/A | 114 (35.6) | 45 (38.1) | Ref. | 0.63 | Ref. | 0.70 |
|  | A/C+C/C | 206 (64.4) | 73 (61.9) | 1.11 (0.72 – 1.72) |  | 0.84 (0.35 – 2.02) |  |
|  | Recessive | | |  |  |  |  |
|  | A/A+A/C | 269 (84.1) | 101 (85.6) | Ref. | 0.70 | Ref. | 0.87 |
|  | C/C | 51 (15.9) | 17 (14.4) | 1.13 (0.62 – 2.04) |  | 1.09 (0.37 – 3.22) |  |
|  | Allelic | | |  |  |  |  |
|  | A | 383 (59.8) | 146 (61.9) | Ref. | 0.59 | Ref. | 0.86 |
|  | C | 257 (40.2) | 90 (38.1) | 1.09 (0.80 – 1.48) |  | 0.95 (0.53 – 1.71) |  |

*** Adjusted by age, gender, ethnicity and body mass index (BMI). A-OR, adjusted odds ratio; CI, confidence interval; C-OR, crude odds ratio.

**Table S4**. Potential effect modifiers in the gene-disease interaction of the *CYP27A1* rs17470271 polymorphism with diabetic kidney disease.

|  | **Genotypic comparison model** | | | | **Dominant model** | | | **Recessive model** | | |
| --- | --- | --- | --- | --- | --- | --- | --- | --- | --- | --- |
|  | **A/A** | **A/T** | **T/T** | ***p*** | **A/A** | **A/T+T/T** | ***p*** | **AA+AT** | **TT** | ***p*** |
| **DKD group** | | | | | | | | | | |
| Age, year, median (IQR) | 64.2 (56.7 – 68.6) | 61.7 (58.0 – 67.6) | 58.8 (57.2 – 59.3) | 0.22 | 64.2 (56.7 – 68.6) | 61.3 (57.3 – 67.6) | 0.33 | 63.9 (57.3 – 67.9) | 58.8 (57.2 – 59.3) | 0.11 |
| Male, *n* (%)  Female, *n* (%) | 43 (56.6) | 31 (40.8) | 2 (2.6) | 0.67 | 43 (56.6) | 33 (43.4) | 0.43 | 74 (97.4) | 2 (2.6) | 0.80 |
|  | 57 (62.6) | 31 (34.1) | 3 (3.3) |  | 57 (62.6) | 34 (37.4) |  | 88 (96.7) | 3 (3.3) |  |
| Kazakh, *n* (%)  Non-Kazakh, *n* (%) | 89 (60.5) | 54 (36.7) | 4 (2.8) | 0.86 | 89 (60.5) | 58 (39.5) | 0.77 | 143 (97.3) | 4 (2.7) | 0.61 |
|  | 12 (57.1) | 8 (38.1) | 1 (4.8) |  | 12 (57.1) | 9 (42.9) |  | 20 (95.2) | 1 (4.8) |  |
| Diabetes duration, median (IQR) | 13.8 (10 - 19) | 14 (9 – 18.8) | 15 (10.1 - 22) | 0.75 | 13.8 (10 - 19) | 14.0 (9.0 – 19.0) | 0.60 | 13.9 (9.6 – 19.0) | 15 (10.1 – 22.0) | 0.68 |
| Family history of diabetes,  *n* (%)  No Family history of diabetes,  *n* (%) | 47 (62.7) | 25 (33.3) | 3 (4.0) | 0.67 | 47 (62.7) | 28 (37.3) | 0.64 | 72 (96.0) | 3 (4.0) | 0.52 |
|  | 52 (59.1) | 34 (38.6) | 2 (2.2) |  | 52 (59.1) | 36 (40.9) |  | 86 (97.7) | 2 (2.3) |  |
| Rural, *n* (%)  Urban *n* (%) | 10 (71.4) | 3 (21.4) | 1 (7.2) | 0.35 | 10 (71.4) | 4 (28.6) | 0.38 | 13 (92.9) | 1 (7.1) | 0.35 |
|  | 89 (59.3) | 57 (38.0) | 4 (2.7) |  | 89 (59.3) | 61 (40.7) |  | 146 (97.3) | 4 (2.7) |  |
| Current smoker, *n* (%)  Former smoker *n* (%)  Never smoker *n* (%) | 11 (55.0) | 8 (40.0 | 1 (5.0) | 0.86 | 11 (55.0) | 9 (45.0) | 0.81 | 19 (95) | 1 (5.0) | 0.58 |
|  | 16 (64.0) | 9 (36.0) | 0 (0.0) |  | 16 (64.0) | 9 (36.0) |  | 25 (100.0) | 0 (0.0) |  |
|  | 72 (61.5) | 41 (35.1) | 4 (3.4) |  | 72 (61.5) | 45 (38.5) |  | 113 (96.6) | 4 (3.4) |  |
| BMI, kg/m^2^, median (IQR) | 29.0 (25.9 – 31.1) | 28.3 (26.5 – 31.1) | 25.5 (23.5 – 33.2) | 0.90 | 29.0 (25.9 – 31.1) | 28.2 (26.2 – 31.2) | 0.79 | 28.7 (26.1 – 31.1) | 25.5 (23.5 – 33.2) | 0.77 |
| Arterial hypertension, *n* (%)  No Arterial hypertension, *n* (%) | 95 (61.7) | 54 (35.1) | 5 (3.2) | 0.39 | 95 (61.7) | 59 (38.3) | 0.29 | 149 (96.8) | 5 (3.2) | 0.54 |
|  | 5 (45.5) | 6 (54.5) | 0 (0.0) |  | 5 (45.5) | 6 (54.5) |  | 11 (100.0) | 0 (0.0) |  |
| Myocardial Infarction, *n* (%)  No Myocardial Infarction, *n* (%) | 17 (56.7) | 12 (40.0) | 1 (3.3) | 0.88 | 17 (56.7) | 13 (43.3) | 0.61 | 29 (96.7) | 1 (3.3) | 0.93 |
|  | 82 (61.7) | 47 (35.3) | 4 (3.0) |  | 82 (61.7) | 51 (38.3) |  | 129 (97.0) | 4 (3.0) |  |
| Stroke, *n* (%)  No Stroke, *n* (%) | 21 (63.6) | 11 (33.3) | 1 (3.1) | 0.93 | 21 (63.6) | 12 (36.4) | 0.70 | 32 (97.0) | 1 (3.0) | 0.99 |
|  | 78 (60.0) | 48 (36.9) | 4 (3.1) |  | 78 (60.0) | 52 (40.0) |  | 126 (97.0) | 4 (3.0) |  |
| ACE inhibitors/ARB, n (%)  No ACE inhibitors/ARB, n (%) | 36 (53.7)  63 (66.3) | 29 (43.3)  29 (30.5) | 2 (3.0)  3 (3.2) | 0.25 | 36 (53.7)  63 (66.3) | 31 (46.3)  32 (33.7) | 0.11 | 65 (97.0)  92 (96.8) | 2 (3.0)  3 (3.2) | 0.95 |
| **Non-DKD group** | | | | | | | | | | |
| Age, year, median (IQR) | 60.7 (55.7 – 65.6) | 62.9 (55.0 – 67.4) | 63.2 (53.0 - 66.0) | 0.89 | 60.7 (55.7 – 65.6) | 63.1 (53.7 – 67.3) | 0.64 | 61.3 (55.7 – 67.3) | 63.2 (53.0 – 66.0) | 0.89 |
| Male, *n* (%)  Female, *n* (%) | 43 (64.2) | 18 (26.9) | 6 (8.9) | 0.99 | 43 (64.2) | 24 (35.8) | 0.99 | 61 (91.0) | 6 (9.0) | 0.91 |
|  | 54 (64.3) | 22 (26.2) | 8 (9.5) |  | 54 (64.3) | 30 (35.7) |  | 76 (90.5) | 8 (9.5) |  |
| Kazakh, *n* (%)  Non-Kazakh, *n* (%) | 78 (62.9) | 35 (28.2) | 11 (8.9) | 0.58 | 78 (62.9) | 46 (37.1) | 0.46 | 113 (91.1) | 11 (8.9) | 0.72 |
|  | 19 (70.4) | 5 (18.5) | 3 (11.1) |  | 19 (70.4) | 8 (29.6) |  | 24 (88.9) | 3 (11.1) |  |
| Diabetes duration, median (IQR) | 12.7 (10.7 – 15.9) | 11.2 (10.3 – 14.9) | 13.4 (9.9 – 14.1) | 0.37 | 12.7 (10.7 – 15.9) | 11.5 (10.2 – 14.9) | 0.18 | 12.3 (10.5 – 15.8) | 13.4 (9.9 – 14.1) | 0.85 |
| Family history of diabetes,  *n* (%)  No Family history of diabetes,  *n* (%) | 53 (61.6) | 22 (25.6) | 11 (12.8) | 0.15 | 53 (61.6) | 33 (38.4) | 0.45 | 75 (87.2) | 11 (12.8) | 0.05 |
|  | 40 (67.8) | 17 (28.8) | 2 (3.4) |  | 40 (67.8) | 19 (32.2) |  | 57 (96.6) | 2 (3.4) |  |
| Rural, *n* (%)  Urban *n* (%) | 12 (63.2) | 4 (21.1) | 3 (15.7) | 0.49 | 12 (63.2) | 7 (36.8) | 0.91 | 16 (84.2) | 3 (15.8) | 0.26 |
|  | 82 (64.6) | 35 (27.6) | 10 (7.8) |  | 82 (64.6) | 45 (35.4) |  | 117 (92.1) | 10 (7.9) |  |
| Current smoker, *n* (%)  Former smoker *n* (%)  Never smoker *n* (%) | 11 (64.7) | 5 (29.4) | 1 (5.9) | 0.53 | 11 (64.7) | 6 (35.3) | 0.65 | 16 (94.1) | 1 (5.9) | 0.21 |
|  | 16 (72.7) | 6 (27.3) | 0 (0.0) |  | 16 (72.7) | 6 (27.3) |  | 22 (100.0) | 0 (0.0) |  |
|  | 66 (62.3) | 28 (26.4) | 12 (11.3) |  | 66 (62.3) | 40 (37.7) |  | 94 (88.7) | 12 (11.3) |  |
| BMI, kg/m^2^, median (IQR) | 30.4 (26.6 – 34.6) | 29.3 (26.1 – 32.5) | 30.1 (27.3 – 32.0) | 0.63 | 30.4 (26.6 – 34.6) | 29.5 (26.4 – 32.0) | 0.38 | 29.6 (26.4 – 34.1) | 30.1 (27.3 – 32.0) | 1.00 |
| Arterial hypertension, *n* (%)  No Arterial hypertension, *n* (%) | 68 (63.6) | 29 (27.1) | 10 (9.3) | 0.95 | 68 (63.6) | 39 (36.4) | 0.81 | 97 (90.7) | 10 (9.3) | 0.79 |
|  | 25 (65.8) | 10 (26.3) | 3 (7.9) |  | 25 (65.8) | 13 (34.2) |  | 35 (92.1) | 3 (7.9) |  |
| Myocardial Infarction, *n* (%)  No Myocardial Infarction, *n* (%) | 9 (75.0) | 3 (25.0) | 0 (0.0) | 0.49 | 9 (75.0) | 3 (25.0) | 0.41 | 12 (100.0) | 0 (0) | 0.26 |
|  | 84 (63.2) | 36 (27.1) | 13 (9.7) |  | 84 (63.2) | 49 (36.8) |  | 120 (90.2) | 13 (9.8) |  |
| Stroke, *n* (%)  No Stroke, *n* (%) | 11 (91.7) | 0 (0) | 1 (8.3) | 0.08 | **11 (91.7)** | **1 (8.3)** | **0.038** | 11 (91.7) | 1 (8.3) | 0.94 |
|  | 82 (61.7) | 39 (29.3) | 12 (9.0) |  | **82 (61.7)** | **51 (38.3)** |  | 121 (91.0) | 12 (9.0) |  |
| ACE inhibitors/ARB, n (%)  No ACE inhibitors/ARB, n (%) | 47 (66.2)  46 (62.2) | 18 (25.4)  21 (28.4) | 6 (8.4)  7 (9.4) | 0.88 | 47 (66.2)  46 (62.2) | 24 (33.8)  28 (37.8) | 0.61 | 65 (91.6)  67 (90.5) | 6 (8.4)  7 (9.5) | 0.83 |

ACE inhibitors, angiotensin-converting enzyme inhibitors; ARBs, angiotensin II receptor blockers; BMI, body mass index.

Boldface indicates statistically significant differences (*p* < 0.05).

**Table S5**. Potential effect modifiers in the gene-disease interaction of the *GC* rs4588 polymorphism with diabetic kidney disease.

|  | **Genotypic comparison model** | | | | **Dominant model** | | | **Recessive model** | | |
| --- | --- | --- | --- | --- | --- | --- | --- | --- | --- | --- |
|  | **G/G** | **G/T** | **T/T** | ***p*** | **G/G** | **G/T+T/T** | ***p*** | **GG+GT** | **TT** | ***p*** |
| **DKD group** | | | | | | | | | | |
| Age, year, median (IQR) | 63.0 (56.9 – 67.4) | 63.4 (56.4 – 68.8) | 64.9 (64.3 – 67.6) | 0.42 | 63 (56.9 – 67.4) | 63.9 (57.6 – 68.8) | 0.62 | 63.2 (56.9 – 67.8) | 64.9 (64.3 – 67.6) | 0.20 |
| Male, *n* (%)  Female, *n* (%) | 47 (61.8) | 26 (34.2) | 3 (4.0) | 0.67 | 47 (61.8) | 29 (38.2) | 0.37 | 73 (96.1) | 3 (3.9) | 0.89 |
|  | 50 (55.0) | 37 (40.6) | 4 (4.4) |  | 50 (55.0) | 41 (45.0) |  | 87 (95.6) | 4 (4.4) |  |
| Kazakh, *n* (%)  Non-Kazakh, *n* (%) | **88 (59.9)** | **56 (38.1)** | **3 (2.0)** | **0.001** | 88 (59.9) | 59 (40.1) | 0.29 | **144 (98.0)** | **3 (2.0)** | **<0.001** |
|  | **10 (47.6)** | **7 (33.3)** | **4 (19.1)** |  | 10 (47.6) | 11 (52.4) |  | **17 (80.9)** | **4 (19.1)** |  |
| Diabetes duration, median (IQR) | 13.9 (9.0 – 19.2) | 15.0 (10.1 – 18.0) | 15.0 (9.7 – 20.0) | 0.87 | 13.9 (9.0 – 19.2) | 15.0 (10.1 – 18.0) | 0.61 | 14 (9.4 - 19) | 15 (9.7 - 20) | 0.87 |
| Family history of diabetes,  *n* (%)  No Family history of diabetes,  *n* (%) | 42 (56.0) | 30 (40.0) | 3 (4.0) | 0.92 | 42 (56.0) | 33 (44.0) | 0.69 | 72 (96.0) | 3 (4.0) | 0.84 |
|  | 52 (59.1) | 33 (37.5) | 3 (3.4) |  | 52 (59.1) | 36 (40.9) |  | 85 (96.6) | 3 (3.4) |  |
| Rural, *n* (%)  Urban *n* (%) | 11 (78.6) | 3 (21.4) | 0 (0.0) | 0.26 | 11 (78.6) | 3 (21.4) | 0.11 | 14 (100.0) | 0 (0.0) | 0.45 |
|  | 85 (56.7) | 59 (39.3) | 6 (4.0) |  | 85 (56.7) | 65 (43.3) |  | 144 (96.0) | 6 (4.0) |  |
| Current smoker, *n* (%)  Former smoker *n* (%)  Never smoker *n* (%) | 12 (60.0) | 7 (35.0) | 1 (5.0) | 0.98 | 12 (60.0) | 8 (40.0) | 0.92 | 19 (95.0) | 1 (5.0) | 0.94 |
|  | 15 (60.0) | 9 (36.0) | 1 (4.0) |  | 15 (60.0) | 10 (40.0) |  | 24 (96.0) | 1 (4.0) |  |
|  | 66 (56.4) | 47 (40.2) | 4 (3.4) |  | 66 (56.4) | 51 (43.6) |  | 113 (96.6) | 4 (3.4) |  |
| BMI, kg/m^2^, median (IQR) | 28.8 (26.0 – 31.6) | 28.1 (25.8 – 30.5) | 29.7 (24.7 – 30.5) | 0.88 | 28.8 (26.0 – 31.6) | 28.2 (25.7 – 30.5) | 0.66 | 28.5 (25.8 – 31.3) | 29.7 (24.7 – 30.5) | 0.88 |
| Arterial hypertension, *n* (%)  No Arterial hypertension, *n* (%) | 87 (56.5) | 61 (39.6) | 6 (3.9) | 0.31 | 87 (56.5) | 67 (43.5) | 0.29 | 148 (96.1) | 6 (3.9) | 0.41 |
|  | 8 (72.7) | 2 (18.2) | 1 (9.1) |  | 8 (72.7) | 3 (27.3) |  | 10 (90.9) | 1 (9.1) |  |
| Myocardial Infarction, *n* (%)  No Myocardial Infarction, *n* (%) | 18 (60.0) | 12 (40.0) | 0 (0.0) | 0.56 | 18 (60.0) | 12 (40.0) | 0.83 | 30 (100.0) | 0 (0.0) | 0.28 |
|  | 77 (57.9) | 51 (38.4) | 5 (3.7) |  | 77 (57.9) | 56 (42.1) |  | 128 (96.2) | 5 (3.8) |  |
| Stroke, *n* (%)  No Stroke, *n* (%) | 19 (57.6) | 14 (42.4) | 0 (0.0) | 0.49 | 19 (57.6) | 14 (42.4) | 0.93 | 33 (100.0) | 0 (0.0) | 0.25 |
|  | 76 (58.5) | 49 (37.7) | 5 (3.8) |  | 76 (58.5) | 54 (41.5) |  | 125 (96.2) | 5 (3.8) |  |
| ACE inhibitors/ARB, n (%)  No ACE inhibitors/ARB, n (%) | 36 (53.7)  57 (60.0) | 29 (43.3)  34 (35.8) | 2 (3.0)  4 (4.2) | 0.61 | 36 (53.7)  57 (60.0) | 31 (46.3)  38 (40.0) | 0.43 | 65 (97.0)  91 (95.8) | 2 (3.0)  4 (4.2) | 0.68 |
| **Non-DKD group** | | | | | | | | | | |
| Age, year, median (IQR) | 62.5 (55.9 – 67.4) | 59.6 (51.4 – 65.3) | 64.5 (60.5 – 67.4) | 0.20 | 62.5 (55.9 – 67.4) | 60.8 (53.0 -66.0) | 0.18 | 61.3 (54.1 – 67.0) | 64.5 (60.5 – 67.4) | 0.53 |
| Male, *n* (%)  Female, *n* (%) | 38 (56.7) | 24 (35.8) | 5 (7.5) | 0.91 | 38 (56.7) | 29 (43.3) | 0.86 | 62 (92.5) | 5 (7.5) | 0.67 |
|  | 47 (55.3) | 30 (35.3) | 8 (9.4) |  | 47 (55.3) | 38 (44.7) |  | 77 (90.6) | 8 (9.4) |  |
| Kazakh, *n* (%)  Non-Kazakh, *n* (%) | **76 (60.8)** | **39 (31.2)** | **10 (8.0)** | **0.031** | **76 (60.8)** | **49 (39.2)** | **0.009** | 115 (92.0) | 10 (8.0) | 0.60 |
|  | **9 (33.3)** | **15 (55.6)** | **3 (11.1)** |  | **9 (33.3)** | **18 (66.7)** |  | 24 (88.9) | 3 (11.1) |  |
| Diabetes duration, median (IQR) | 12.1 (10.5 – 15.9) | 11.9 (10.2 – 14.3) | 14.9 (10.9 – 16.7) | 0.37 | 12.1 (10.5 – 15.9) | 12.5 (10.2 – 16.0) | 0.98 | 12.1 (10.4 – 15.8) | 14.9 (10.9 – 16.7) | 0.19 |
| Family history of diabetes,  *n* (%)  No Family history of diabetes,  *n* (%) | 46 (53.5) | 34 (39.5) | 6 (7.0) | 0.53 | 46 (53.5) | 40 (46.5) | 0.86 | 80 (93.0) | 6 (7.0) | 0.33 |
|  | 33 (55.0) | 20 (33.3) | 7 (11.7) |  | 33 (55.0) | 27 (45.0) |  | 53 (88.3) | 7 (11.7) |  |
| Rural, *n* (%)  Urban *n* (%) | 11 (57.9) | 8 (42.1) | 0 (0.0) | 0.34 | 11 (57.9) | 8 (42.1) | 0.75 | 19 (100.0) | 0 (0.0) | 0.15 |
|  | 69 (53.9) | 46 (35.9) | 13 (10.2) |  | 69 (53.9) | 59 (46.1) |  | 115 (89.8) | 13 (10.2) |  |
| Current smoker, *n* (%)  Former smoker *n* (%)  Never smoker *n* (%) | 7 (41.2) | 7 (41.2) | 3 (17.6) | 0.34 | 7 (41.2) | 10 (58.8) | 0.23 | 14 (82.4) | 3 (17.6) | 0.39 |
|  | 15 (68.2) | 5 (22.7) | 2 (9.1) |  | 15 (68.2) | 7 (31.8) |  | 20 (90.9) | 2 (9.1) |  |
|  | 57 (53.3) | 42 (39.3) | 8 (7.4) |  | 57 (53.3) | 50 (46.7) |  | 99 (92.5) | 8 (7.5) |  |
| BMI, kg/m^2^, median (IQR) | 29.6 (26.6 – 34.5) | 30.3 (26.2 – 34.7) | 27.8 (26.0 – 31.9) | 0.48 | 29.6 (26.6 – 34.5) | 29.4 (26.1 – 34.1) | 0.80 | 29.7 (26.5 – 34.5) | 27.8 (26.0 – 31.9) | 0.23 |
| Arterial hypertension, *n* (%)  No Arterial hypertension, *n* (%) | 59 (54.6) | 39 (36.1) | 10 (9.3) | 0.92 | 59 (54.6) | 49 (45.4) | 0.83 | 98 (90.7) | 10 (9.3) | 0.80 |
|  | 20 (52.6) | 15 (39.5) | 3 (7.9) |  | 20 (52.6) | 18 (47.4) |  | 35 (92.1) | 3 (7.9) |  |
| Myocardial Infarction, *n* (%)  No Myocardial Infarction, *n* (%) | 6 (50.0) | 6 (50.0) | 0 (0.0) | 0.41 | 6 (50.0) | 6 (50.0) | 0.77 | 12 (100.0) | 0 (0.0) | 0.26 |
|  | 73 (54.5) | 48 (35.8) | 13 (9.7) |  | 73 (54.5) | 61 (45.5) |  | 121 (90.3) | 13 (9.7) |  |
| Stroke, *n* (%)  No Stroke, *n* (%) | **3 (25.0)** | **6 (50.0)** | **3 (25.0)** | **0.04** | **3 (25.0)** | **9 (75.0)** | **0.035** | **9 (75.0)** | **3 (25.0)** | **0.041** |
|  | **76 (56.7)** | **48 (35.8)** | **10 (7.5)** |  | **76 (56.7)** | **58 (43.3)** |  | **124 (92.5)** | **10 (7.5)** |  |
| ACE inhibitors/ARB, n (%)  No ACE inhibitors/ARB, n (%) | 42 (58.3)  37 (50.0) | 24 (33.3)  30 (40.5) | 6 (8.4)  7 (9.5) | 0.60 | 42 (58.3)  37 (50.0) | 30 (41.7)  37 (50.0) | 0.31 | 66 (91.7)  67 (90.5) | 6 (8.3)  7 (9.5) | 0.81 |

ACE inhibitors, angiotensin-converting enzyme inhibitors; ARBs, angiotensin II receptor blockers; BMI, body mass index.

Boldface indicates statistically significant differences (*p* < 0.05).

**Table S6.** Correlation analysis between *CYP27A1* rs17470271 and *GC* rs4588 polymorphisms with demographic and clinical characteristics.

| **Variables** | **rs17470271 *[CYP27A1]*** | | | **rs4588 *[GC]*** | | |
| --- | --- | --- | --- | --- | --- | --- |
|  | **Total** | **DKD** | **non-DKD** | **Total** | **DKD** | **non-DKD** |
| Age, years | –0.02 (0.68) | –0.09 (0.25) | 0.03 (0.65) | –0.02 (0.78) | 0.05 (0.49) | –0.08 (0.39) |
| Gender | –0.02 (0.61) | –0.06 (0.47) | 0.001 (0.99) | 0.05 (0.42) | 0.07 (0.38) | 0.02 (0.80) |
| Ethnicity | 0.01 (0.83) | –0.03 (0.72) | 0.05 (0.58) | **–0.17 (0.003)** | –0.12 (0.11) | **–0.20 (0.02)** |
| Diabetes duration, years | –0.06 (0.33) | –0.03 (0.66) | –0.10 (0.22) | 0.05 (0.43) | 0.04 (0.61) | 0.03 (0.72) |
| Family history of diabetes | 0.02 (0.63) | –0.02 (0.72) | 0.09 (0.28) | 0.02 (0.72) | 0.03 (0.68) | –0.007 (0.92) |
| Place of living | 0.01 (0.84) | 0.06 (0.48) | –0.03 (0.72) | 0.08 (0.14) | 0.13 (0.10) | 0.05 (0.52) |
| Marrital status | –0.04 (0.47) | 0.10 (0.19) | **–0.19 (0.03)** | 0.02 (0.78) | 0.12 (0.13) | –0.08 (0.31) |
| Smoking status | –0.04 (0.44) | 0.0001 (0.99) | –0.09 (0.27) | –0.03 (0.63) | –0.03 (0.74) | –0.03 (0.77) |
| BMI, kg/m^2^ | –0.02 (0.72) | 0.02 (0.83) | –0.06 (0.44) | –0.03 (0.62) | –0.03 (0.69) | –0.04 (0.60) |
| Arterial hypertension | –0.01 (0.86) | –0.07 (0.35) | 0.02 (0.79) | 0.003 (0.96) | 0.07 (0.41) | –0.01 (0.90) |
| Myocardial Infarction | –0.007 (0.91) | 0.04 (0.62) | –0.08 (0.34) | –0.03 (0.65) | –0.03 (0.73) | –0.002 (0.99) |
| Stroke | –0.07 (0.19) | –0.03 (0.72) | **–0.16 (0.048)** | 0.06 (0.29) | –0.006 (0.95) | **0.20 (0.01)** |
| ACE inhibitors/ARB | 0.04 (0.47) | 0.12 (0.12) | –0.04 (0.62) | –0.008 (0.89) | 0.05 (0.49) | –0.08 (0.34) |

Results are presented as Spearman’s rank correlation coefficients (*p*-value). ACE inhibitors, angiotensin-converting enzyme inhibitors; ARBs, angiotensin II receptor blockers; BMI, body mass index.

Boldface indicates statistically significant differences (*p* < 0.05).

**Table S7.** Haplotype-based analysis of *GC* variants with susceptibility to diabetic kidney disease (DKD): comparison between individuals with and without DKD.

| **Block** | **rs4588 *[GC]*** | **rs7041 *[GC]*** | **DKD, frequency** | **non-DKD, frequency** | **OR (95% CI)** | ***p*-value** |
| --- | --- | --- | --- | --- | --- | --- |
| 1 | G | C | 0.41 | 0.39 | Ref. | – |
| 2 | G | A | 0.36 | 0.34 | 0.97 (0.68 - 1.38) | 0.85 |
| 3 | T | A | 0.23 | 0.26 | 0.85 (0.57 - 1.27) | 0.43 |
| 4 | T | C | 0 | 0.01 | – | – |

CI, confidence interval; DKD, diabetic kidney disease; OR, crude odds ratio.
